# Monkeypox: an in-depth examination of epidemiology, pathophysiology, and global public health implications

**DOI:** 10.1097/MS9.0000000000003064

**Published:** 2025-02-27

**Authors:** Khaled Moghib, Awaish Asim, Izere Salomon, Thoria I. Ghanm, Trisha Shivashankar

**Affiliations:** a Faculty of Medicine, Cairo University, Cairo, Egypt; b Medical Research Group of Egypt, Negida Academy, Arlington, Massachusetts, USA; c Samarkand State Medical University, Samarkand, Uzbekistan; d University of Rwanda College of Medicine and Health Sciences, Kigali, Rwanda; e Faculty of Medicine, Mansoura University, Mansoura, Egypt; f Bharati Vidyapeeth Medical College, Pune, India

**Keywords:** antiviral therapy, epidemiology, monkeypox virus, mpox, outbreak management, public health, vaccination, zoonotic disease

## Abstract

**Background:**

Mpox, formerly known as monkeypox, is a zoonotic disease caused by the monkeypox virus (MPXV) within the *Orthopoxvirus* genus. Following the cessation of smallpox vaccination, mpox has re-emerged as a significant public health concern, exacerbated by global outbreaks in non-endemic regions.

**Objectives:**

This narrative review aims to explore the epidemiology, transmission patterns, clinical manifestations, treatment options, and public health implications of mpox. It seeks to identify research gaps and propose strategies for prevention and management.

**Methodology:**

A comprehensive review of the literature was conducted using scientific databases and credible health organization reports. Inclusion criteria focused on studies addressing mpox epidemiology, pathophysiology, clinical presentations, and treatment strategies published up to 2024.

**Results:**

Mpox has transitioned from zoonotic origins to significant human-to-human transmission, particularly via sexual contact. Outbreaks have highlighted diverse clinical presentations, including dermatological, respiratory, and systemic symptoms, with immunocompromised individuals disproportionately affected. Tecovirimat and JYNNEOS vaccines show promise, but challenges in vaccine distribution and underreporting persist. Public health responses remain uneven, with significant disparities in low-resource settings.

**Conclusion:**

Mpox remains a pressing global health concern requiring robust surveillance, equitable vaccine access, and community-centered awareness campaigns. Research priorities include genomic studies, improved diagnostics, and targeted therapeutic approaches to mitigate future outbreaks and their socioeconomic impact.

## Introduction

Mpox is an infectious and contagious zoonotic disease caused by mpox virus (MPXV), which belongs to the genus Orthopoxvirus. Poxviruses, which are large DNA viruses, differ from smaller viruses in their strategies for overcoming immunological barriers^[1]^. MPXV falls into two distinct clades based on genetic and geographic variations: former Central Africa (or Congo Basin) clade, now known as clade I, and the former West African clade, now known as clade II. There is an approximately 0.5% genomic sequence difference between the two clades, with the former appearing more virulent based on the higher observed mortality rates^[2]^.

**Highlights**

- Reviews over 102 997 confirmed monkeypox cases and 223 deaths globally as of July 2024, highlighting the dramatic rise during the 2022–2024 outbreaks.
- Examines transmission dynamics, noting that 85.5% of cases occur in men who have sex with men (MSM) and emphasizing zoonotic and human-to-human spread.
- Evaluate treatment options including tecovirimat and brincidofovir, and discuss the use of smallpox vaccines for post-exposure prophylaxis amid the lack of specific monkeypox vaccines.
- Calls for strengthened global surveillance and public health strategies following the WHO’s declaration of monkeypox as a Public Health Emergency of International Concern (PHEIC) in July 2022.
- Identifies significant research gaps, advocating for enhanced diagnostics and collaborative efforts to improve understanding of monkeypox transmission, prevention, and treatment.

## Historical context and prevalence

**Figure 1.**
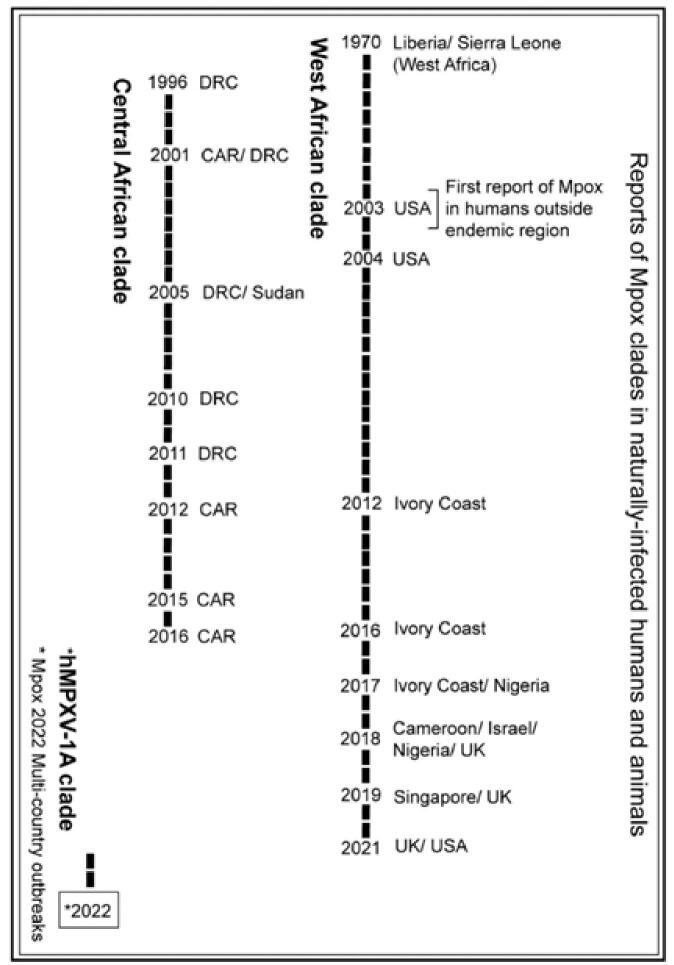
Timeline illustrating key monkeypox outbreaks, highlighting geographic distribution and epidemiological milestones.

Mpox was discovered in 1958 and initially documented in humans in 1970. Before 2017, mpox mainly occurred in central and western Africa, where there was ongoing MPXV transmission in local animal hosts and occasional spillover into human communities, predominantly in rural regions. The initial zoonotic case of human mpox was documented in the Democratic Republic of the Congo (DRC) in 1970. The virus was traced back to monkeys in a Danish laboratory, where it was first identified as the causative agent of a pox-like illness in 1959^[1]^. In 1967, WHO launched collaborative epidemic-epidemiological surveillance to carry out serological surveys, track mpox epidemics, and assess the geographic source range of the virus (Fig. 1)^[3]^.

### Early identification and historical outbreaks

The first human mpox outbreak occurred between 1970 and 1971, with a 9-month-old infant being the first recorded case admitted to Basankusu Hospital in the DRC on 1 September 1970 (Fig. 1)^[4]^. Following this case, sporadic outbreaks were reported primarily among children in the rural rainforests of Central and West Africa, particularly in the DRC, where high fatality rates (1%–2%) were noted. However, many of these cases lacked laboratory confirmation owing to limited local diagnostic capabilities and challenges related to civil unrest^[5,6]^. The first major outbreak outside Africa took place in the United States in 2003, stemming from a 3-year-old girl who was bitten by an infected prairie dog imported from Ghana alongside other rodents. Suspected and confirmed cases were documented in the Center for Disease Control and Prevention (CDC), including 71 cases^[7]^.

### Global spread and recent outbreaks

Although mpox has infected Western and Central Africans for five decades, it has not attracted enough preventive and therapeutic interventions to prevent an epidemic. The first outbreak of human mpox outside its endemic region, registered in the USA, was indirectly related to various infected rodent species imported from West Africa. This outbreak occurred in prairie dogs that were in contact with rodent species imported from Ghana. Consequently, prairie dogs transmitted MPXV to approximately 40 humans. This was the first known outbreak of MPOX in Africa. Before 2022, the information accessible regarding mpox was related to incidents in Africa, centered on an outbreak in a single nation associated with the importation of infected small animals, along with several cases reported during travel from 2018 to 2021. Beginning with the 2022–2023 multicountry outbreak, numerous studies have examined the epidemiological and clinical features of mpox cases. These studies tended to be smaller in scale and were frequently confined to regional, national, or local settings. From January 2022 to January 2023, more than 84 110 mpox instances were identified in 110 countries. This has led to an increased incidence of cases, which is now a growing international health concern^[8]^. As of October 2022, all of these transmission events have caused more than seventy thousand cases globally; therefore, the virus has emerged as a threat to spread in regions that did not originate from^[7,9]^.

## Epidemiology

Mpox is a zoonotic disease that has been previously confined to Central and Western Africa, especially in the Democratic Republic of Congo. Although it has emerged in association with the interaction between humans and monkeys, the circumstances today indicate that the natural host is an African rodent. These infections have been reported in squirrels, rats, mice, prairie dogs, and humans. The identification of mpox DNA in semen samples, coupled with the reported isolation of replication-competent mpox from seminal fluid, indicates the potential for sexual transmission of mpox among humans; however, additional research is necessary. The recorded human-to-human household transmission of mpox is believed to occur because of prolonged close contact among patients. The 2022 multicountry outbreaks of mpox were primarily characterized by human-to-human transmission through sexual activities, as noted in 18 studies, raising alarms about the potential of mpox as a sexually transmitted infection. The coinfection of sexually transmitted infections was thoroughly examined during the outbreak. In 2024, the DRC saw its largest registered outbreak of clade I MPXV with over 21 000 suspected cases and up to 700 fatalities. This outbreak features a group of cases linked to sexual transmission of the clade I virus, raising fears of extensive spread via social and sexual interactions akin to the clade IIb outbreak in 2022, primarily among heterosexual networks, although transmission among gay, bisexual, and other men who have sex with men has also been reported^[10]^. Transmission from animals to humans can occur through direct contact with infected animals during grooming or handling of the animals or through bites or scratches^[11]^.

### Incidence and prevalence

The estimation of prevalence and incidence is problematic because of possible underreporting and confirmation problems. However, both have been rising since regular smallpox vaccination stopped^[10]^. Human MPXV has two acknowledged subclasses, originating from different geographical zones across Africa. Both clades can produce rashes similar to smallpox, although clade I, which is present in Central Africa, leads to more severe disease than clade II, which is present in West Africa. Mpox primarily circulates in Central and West Africa, where transmission occurs among animals (mainly primates and rodents), between animals and humans, and through human-to-human interactions. In recent years, swift globalization, population movement, and expanding trade networks have played a role in the international spread of mpox, leading to global outbreaks. Notably, in 2022, a worldwide outbreak of MPOX affected 110 countries and regions. Between 2022 and 2023, 25 503 Mpox cases and 71 fatalities were recorded in Argentina, Brazil, Chile, Colombia, Mexico, and Peru. A notable majority (91.8%–98.5%) were men, with an average age range of 32–35 years. The statistics for each country are as follows: Argentina (2.63; 0.85 to 5.39), Brazil (3.13; 2.61 to 3.69), Chile (2.91; 1.55 to 4.70), Colombia (3.15; 2.07 to 4.44), Mexico (2.28; 1.18 to 3.75), and Peru (2.84; 2.33 to 3.40)^[12]^.

### Demographic factors

Clade I of MPXV was predominantly reported before 2022, and mainly in Central Africa. Some countries that confirmed cases included the Central African Republic, Cameroon, the Republic of the Congo, Gabon, and DR Congo, which were accountable for the most Clade I mpox cases all over the world. In contrast, clade II MPXV infections occurred until the spillover in Nigeria in 2017 and the subsequent leaps in 2022. As of 6 July 2023, a total of 88 122 cases and 148 fatalities had been documented by 112 nations and regions, including 1496 confirmed cases in Canada. In December 2023, the Centers for Disease Control and Prevention (CDC) released a Health Advisory through the Health Alert Network to inform health departments and clinicians about human-to-human transmission of Clade I MPXV associated with sexual activity in the Democratic Republic of the Congo (DRC). Because of restricted diagnostic capabilities and the scarcity of point-of-care tests, the diagnosis of Clade I ox in regions that are most impacted is typically based on clinical findings. The WHO states that insufficient awareness of the virus is a key barrier to stopping the resurgence of MPXV. The ongoing MPXV outbreak in countries where it is not endemic necessitates rigorous epidemiological monitoring to curb the spread of the outbreak to other non-endemic countries. Clade 1b is marked by increased transmissibility, especially via human-to-human interactions, including sexual transmission. This variant has quickly spread from its origin in the Democratic Republic of the Congo to nearby African nations and, further, with verified cases in Europe and Asia. The worldwide reaction has centered on strengthening monitoring, advancing diagnostic methods, and boosting the availability of the Jynneos vaccine and antiviral therapies, such as tecovirimat. Nonetheless, numerous challenges persist, particularly in environments with limited resources. This analysis highlights the necessity for strong public health systems, global cooperation, and continuous research to reduce the effects of mpox and prepare for potential future outbreaks^[13]^.

### Geographic distribution

**Figure 2.**
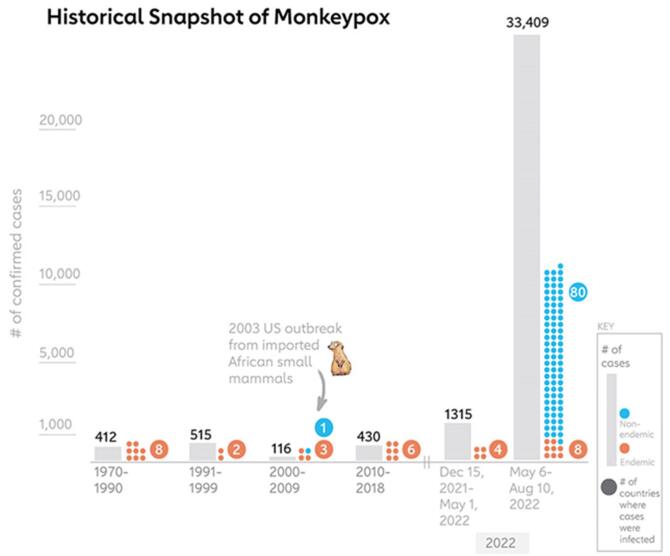
Historical analysis showcasing monkeypox cases’ regional spread and evolution over time.

Although mpox has been observed in different parts of the globe, it mostly affects Africa. The chronicity of the disease is evident in several countries, and its impact is evidenced in morbidity and mortality across the continent, which emphasizes the importance of understanding the disease within the African context^[14]^. The international mpox outbreak in May 2022 could be a new step; it reached Europe, and it became the largest outbreak in Europe, North America, and Asia. The World Health Organization coined this term as an Interim Public Health Danger in July 2022^[15]^. Previously, mpox was documented across several Central and West African countries, including the Central African Republic, the DRC, Liberia, Nigeria, and Sierra Leone (Fig. 2)^[16]^.

## Risk factors

### Zoonotic transmission

Being bitten or coming into contact with an infected animal’s bodily fluids are the two main ways of catching the monkeypox virus (MPXV). Another way to achieve this is to cook bushmeat, which is wild animal meat that is uncooked or processed very little. The reservoir is unknown; however, rodents are likely candidates, whereas humans and monkeys are merely incidental hosts^[17]^.

### Human-to-human transmission

It can occur through the direct method, which is mainly through indirect sexual activity methods, for example, shared clothing, towels, and bedding respiratory routes. Droplet transmission, which could play a role in vertical transmission, can also lead to congenital monkeypox, stillbirth, and miscarriage^[18-20]^.

### Socioeconomic factors

The disease was reported more commonly in younger male individuals, especially in men having sex with men and men with positive and known HIV status^[21,22]^.

## Mechanisms of action

### Viral structure and replication

Monkeypox virus is a dsDNA virus of the orthopoxvirus genus in the family Poxviridae with a brick-like virion. MPXV replicates in the cytoplasm of host cells. Monkeypox proceeds through three phases: virus attachment, hemifusion, and core entry, which occur at the cell membrane or during endocytosis. The transcription process begins with the virally encoded multi-subunit DNA-dependent RNA polymerase and continues with the translation of early, intermediate, and late proteins by host ribosomes. Viruses rely on host-derived transcription factors to complete late and intermediate phases of gene transcription. Synthesis of poxvirus DNA in cytoplasm.^[23-25]^

### Immune evasion

Similar to all orthopoxviruses, the genes that control viral replication, transcription, assembly, and release are cautiously placed in the core region of the genome. The majority of genes that encode host tropism and virulence are located at both ends of the genome. These terminal genes contribute to immune evasion by disrupting apoptosis, antigen presentation and recognition, and signal transduction^[26-29,30]^.

### Clinical manifestations

Nearly 80% of monkeypox patients had no symptoms, 90% had a rash, and 65% had arthritis. Cutaneous lesions first develop in the facial or anogenital area before centrifugally spreading to the trunk, hands, legs, and feet. Forty-one percent of monkeypox patients had mucosal lesions, 73% had a rash in the anogenital area, and 95% had a rash at presentation. A small proportion of patients had a single genital lesion. In addition, the lesions frequently cause pain. On the other hand, the exanthematous rash begins as a macular rash and subsequently as vesicular (umbilicated), papular, and pustular phases. Crusting occurs approximately three weeks after scabbing. Additionally, all the rashes in each body area were in the same stage of development, and 80% of the patients had between 5 and 100 lesions spread throughout various body parts^[27-29]^.

Patients infected with monkeypox are known to experience respiratory symptoms, such as coughing, dyspnea, and nasal congestion. However, secondary bacterial infections of the respiratory system, such as *Streptococcus pneumoniae* and *Mycoplasma pneumoniae*, have been linked to bronchopneumonia and pharyngeal inflammation (pharyngitis), which can cause respiratory discomfort and difficulty breathing. Fever is one of the most commonly reported signs of monkeypox. The degree varied between 38.5°C and 40.5°C, and the fever started 10–14 days after exposure, 1–5 days before the skin rash appeared (12–16 days after exposure). A suspected case of monkeypox with fever and skin lesions was promptly isolated in negative air pressure rooms and intensively monitored for additional symptoms in affluent nations with larger facilities and higher isolation standards. Another common symptom observed in 90% of patients is lymphadenopathy or enlargement of the lymph nodes, which is linked to subsequent bacterial infections. Lymphadenopathy typically appears in the early stages of the infection, 1–2 days after the onset of fever, and can be used as a characteristic to differentiate patients with monkeypox from those with smallpox. Patients have also reported other common nonspecific physical symptoms such as headache, myalgia (muscle pain), back pain, and exhaustion, which typically begin with the onset of fever. In very sick individuals, encephalitis is an uncommon consequence documented during the 2003 outbreak in the United States. This may also occur in vaccinated individuals as a side effect of the shot. Another uncommon side effect of the virus that typically manifests in the second week of infection is the involvement of the gastrointestinal system. It involves diarrhea and vomiting, which dehydrate people and worsen their general health^[31-33]^.

Rather than the MPXV, secondary bacterial infections cause conjunctivitis and eyelid edema. With the right antibiotics, they can be cured within a typical response rate. Some individuals also experience keratitis and corneal ulceration, which result in opacity and visual loss. Fortunately, the use of antimicrobials causes the symptoms to disappear quickly^[34]^.

## Available treatments

Due to their shared genetic background, smallpox antiviral medications, including brincidofovir, tecovirimat, and cidofovir, may be effective against monkeypox. However, currently, there is no cure for monkeypox. CDC permits the use of vaccinia immunoglobulin intravenous (VIGIV) for treating monkeypox during an epidemic. Numerous investigations have documented that patients are administered VIGIV to treat orthopoxvirus infections^[35-37]^.

### Antiviral therapies

Tecovirimat specifically targets VP37 and hinders the intracellular dissemination of viruses by impeding their exit from infected cells. Cidofovir” CDV”, a prodrug, requires phosphorylation by cytoplasmic enzymes upon entering host cells to form CDV diphosphate (CDV-pp), which increases its half-life, suppresses the viral DNA polymerase during DNA replication, and effectively blocks the activity of DNA polymerase 3′–5′ exonuclease^[35,36]^.

### Vaccination strategies

Currently, no vaccination has been developed to prevent infection with MPXV. Immunological cross-protection among orthopoxviruses has led to the recommendation of smallpox vaccinations (vaccinia virus-based) for the current monkeypox epidemics. Furthermore, in its provisional recommendations for monkeypox vaccinations, the World Health Organization (WHO) advised that health workers at risk, clinical laboratory staff conducting diagnostic testing, laboratory personnel dealing with orthopoxviruses, and other individuals at risk under the national policy should ideally receive post-exposure prophylaxis (PEP) within 4 days of initial exposure. The Public Health Agency of Canada (PHAC) has approved IMAMUNE for immunization against monkeypox and orthopox viruses. Similarly, the European Medicine Agency (EMA) recommends IMVANEX for the prevention of monkeypox.^[38-40]^

### Public health interventions

To prevent monkeypox, the CDC recommends avoiding close skin-to-skin contact with individuals displaying monkeypox-like rashes, avoiding contact with objects and materials used in the case of monkeypox, using alcohol-based hand sanitizers before eating or touching the face, and washing hands regularly after using the bathroom^[41]^

## Current prevalence

### Global overview

**Figure 3.**
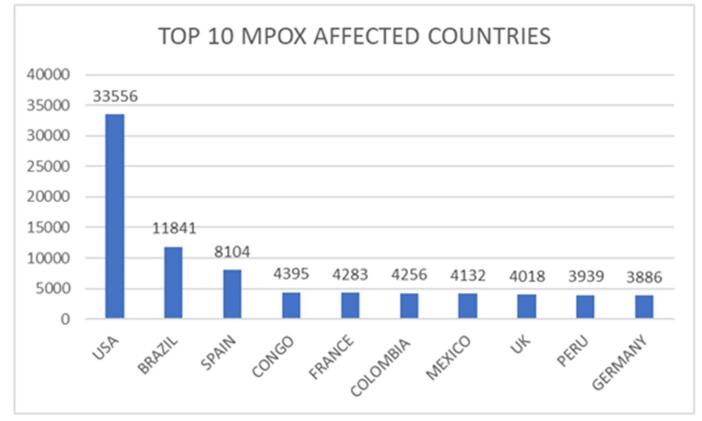
Global distribution of monkeypox cases and fatalities in the top 10 most affected countries (2024)^[3]^.

Mpox, previously known as monkeypox, is endemic to Africa and is caused by the MPXV. Following the 2022 outbreak, reports of mpox cases increased dramatically worldwide^[41]^. The World Health Organization (WHO) Director-General declared the Public Health Emergency of International Concern (PHEIC) due to the rapid spread of a new viral clade 1b. While clades 1a and 2 are also proliferating in numerous African nations, the emergence of clade 1b has accelerated global dissemination of the virus. In addition to Africa, mpox cases have recently been reported in Sweden, Thailand, and Pakistan. Moreover, the demographic groups affected by the virus are evolving and diverse regions encounter distinct challenges. Effective engagement and protection of affected communities are imperative as our comprehension of the disease and its transmission dynamics continues to advance^[40]^. Since the 2022 mpox outbreak, several cases of mpox have been reported, with a overall of 102997 laboratory-confirmed cases and 186 suspected cases as of 31 July 2024. This includes 223 reported deaths, as per the WHO. Most of the cases reported in the last few months have been reported in Central Africa and America. On 1 January 2022, the United States, Brazil, and Spain emerged as the countries most significantly impacted by the mpox (Fig. 3). Collectively, these nations accounted for approximately 80% of global cases reported during this period. Following these three, the Democratic Republic of Congo, France, Colombia, Mexico, the United Kingdom, Peru, and Germany were also heavily affected, each reporting a substantial number. Together, these countries account for 80.0% of the cases reported globally^[40-42]^.

### Case statistics

According to the 2022–2024 Mpox Global Trends report, 102 997 laboratory-confirmed cases and 223 deaths were reported in 121 countries worldwide. In July 2024, 121 cases were confirmed, resulting in 6 fatalities, and 35 countries reported infections^[42]^.

**Figure 4.**
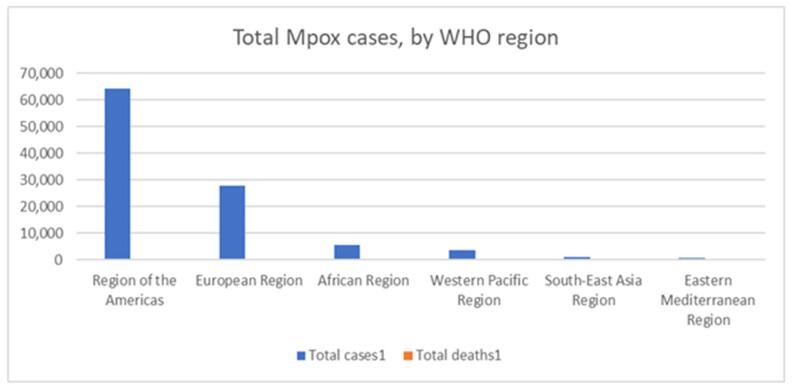
Distribution of new cases reported in July by WHO regions (2024).

**Figure 5.**
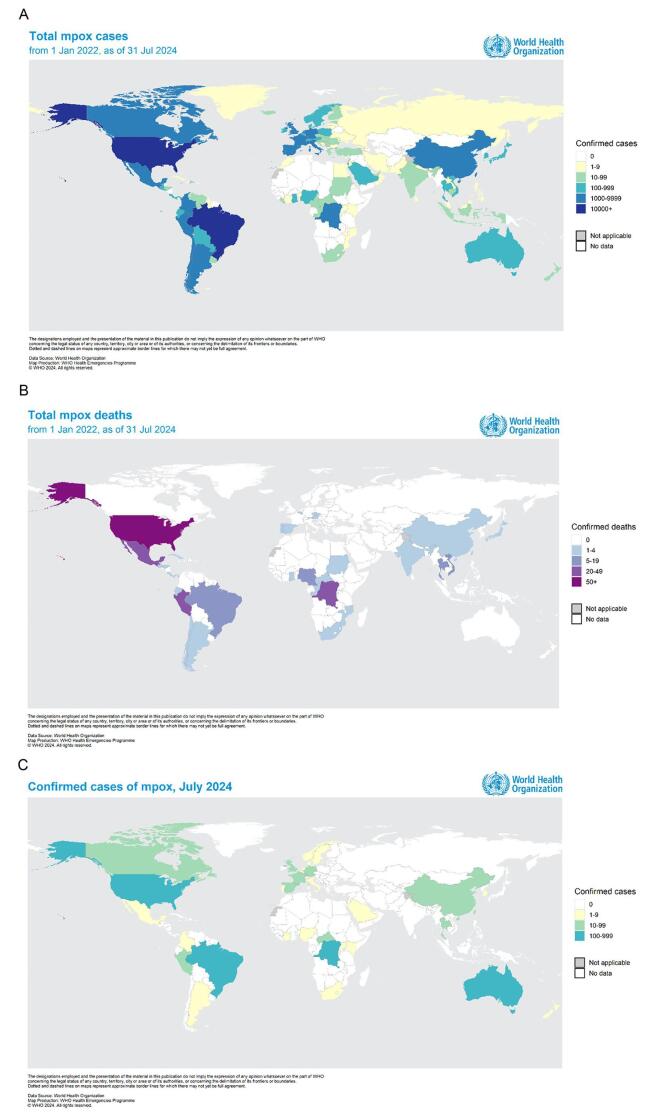
(A, B, and C) Global trends of mpox cases and deaths from 2022 to 2024 based on WHO reports.

A regional analysis of laboratory-confirmed mpox cases in 2024 (Fig. 5A, B, C) revealed a predominance of cases in the DRC, with 3244 cases reported across clades 1a and 1b. Other countries with significant caseloads included Burundi (313 cases, Clade 1b), Nigeria (48 cases, Clade 2), and Central African Republic (45 cases, Clade 1a). South Africa, Congo, Liberia, Uganda, Kenya, and Morocco also reported confirmed mpox cases in varying numbers and clades. The total number of cases from 1 January 2024, to 1 September 2024, was 6154 with almost 3751 cases from 5 August 2024. Further analysis of suspected mpox cases revealed a significant cluster in the DRC, with 1109 confirmed cases. Burundi reported 608 cases, indicating a potential regional outbreak^[42]^. The region-wise confirmed cases of mpox and deaths in America are as follows (Fig. 3): United States of America (33556 cases and 60 deaths), Brazil (11841 cases and 15 deaths), Colombia (4256 cases), Mexico (4132 cases and 34 deaths), Peru (3939 cases and 21 deaths), Canada (1577 cases), Chile (1449 cases and 4 deaths), Argentina (1154 cases and 2 deaths), Ecuador (728 cases and 3 deaths), Guatemala (405 cases and 1 death), Bolivia (265 cases), Panama (245 cases and 1 death), and Costa Rica (226 cases and 1 death). The remaining regions of America constituted 412 cases and 4 deaths, respectively. A similar survey of confirmed mpox cases in the European region is as follows: Spain (8104 cases and 3 deaths), France (4283 cases), The United Kingdom (4018 cases), Germany (3886 cases), the Netherlands (1308 cases), Portugal (1196 cases and 3 deaths), Italy (1056 cases), Belgium (811 cases), Switzerland (579 cases), Austria (348 cases and 1 death), Israel (317 cases), Sweden (300 cases), Ireland (249 cases), Poland (223 cases), Denmark (203 cases), Norway (107 cases), and the remaining regions reported 696 cases with no reported deaths. A total of 864 confirmed mpox cases were reported in the Eastern Mediterranean region, with Saudi Arabia accounting for 763 cases and 9 deaths. In Southeast Asia, 940 cases have been reported, including 820 cases and 15 deaths in Thailand. India and Indonesia were the countries with the highest reported cases. The new cases reported in July included 810 cases from the African region, 345 from the American region, 163 from the Western Pacific region, 154 from the European region, 15 from Southeast Asia, and 9 from the Eastern Mediterranean region (Fig. 4).

### Demographic trends

**Figure 6.**
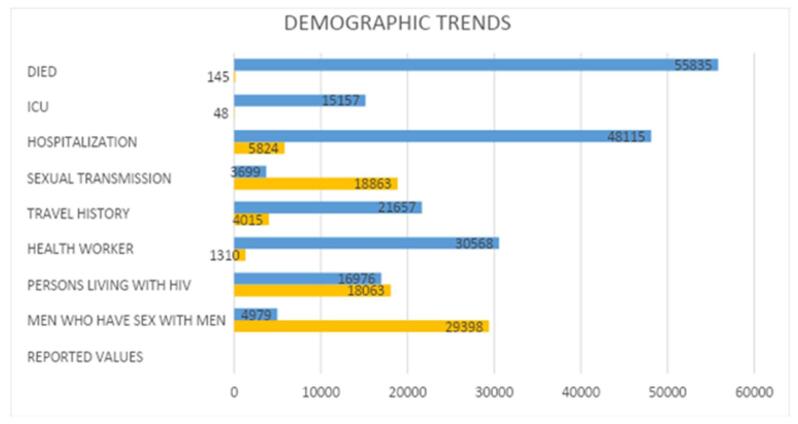
Demographic and risk factor breakdown of global monkeypox cases (2024).

The predominant demographic for confirmed mpox cases was adult males, with a median age of 34 years (Fig. 6). A significant majority of cases (85.5%) occurred among men who had sex with other men. Sexual transmission was the most frequent mode of infection, accounting for 83.6% of reported transmission events. Other notable findings include a higher prevalence among individuals living with HIV (51.6%) and a small proportion of cases^[43]^ involving pregnant or recently pregnant individuals (Fig. 6).

### Factors influencing prevalence

The global reporting of confirmed mpox cases has been hindered by factors such as public awareness and financial constraints. However, since the World Health Organization declared the Public Health Emergency of International Concern, there has been a notable increase in the number of case reports from various countries. In the African Region, the risk factors for mpox clusters include rural and forest dwellings, exposure to bushmeat, and proximity to infected individuals. Additionally, sexual transmission, particularly among men who have sex with men and individuals living with HIV, has been a significant driver for the spread of mpox^[44]^. Several explanations have been reported for the increase in the incidence of mpox^[45]^ as follows:

- The discontinuation of smallpox vaccination in 1980 and the discontinuation of measles and yellow fever vaccinations^[46]^ led to a decline in population-level immunity against *orthopoxviruses*.
- Frequent or prolonged interaction with reservoir species due to displacement of animals from their habitat due to deforestation, urbanization, and consumption of animal meat due to poverty^[47]^
- Increased transmission from humans to humans is due to lack of vaccination, increased virulence, and evolution of the MPXV ^[48]^

Increased reporting of cases due to improvements in diagnostic techniques and decreased awareness of mpox^[49]^

The preponderance of current data relies on passive surveillance, which frequently results in underreporting, potentially obscuring the true extent of human MPXV infections. The other limitations affecting the reporting of the true prevalence of the disease are lack of access to remote regions, lack of manpower to utilize the diagnostic capacity, and financial burden on the population^[50]^. While there has been a clear increase in monkeypox incidence, detailed information is lacking to assess changes in human-to-human transmission rates, morbidity and mortality, and patterns of disease spread^[45]^.

### Surveillance and reporting

Surveillance of mpox is crucial for public health, as it enables the analysis and interpretation of health data to describe health events and identify risks, thus helping in the early detection of outbreaks and effective prevention and control strategies. The importance of diagnosis and surveillance lies in the problem faced by the similar clinical presentations of mpox and herpes viruses^[51]^.

Methods of mpox detection, such as virus isolation from clinical specimens and immunochemistry, require specialized expertise, training, and advanced laboratories, which could lead to underreporting in several mpox–endemic countries, especially those with limited laboratory infrastructure. It was also found that there was severe underrepresentation of mpox in European countries that had more societal stigma towards the 2SLGBTQIAP+ community and comparatively limited access to the testing of sexually transmitted disease and other sexual health services. This has major practical implications, in that, considering the significant underestimation of mpox, it is imperative to take proactive measures to address the issue of health disparities and gaps in the 2SLGBTQIAP+ community^[14]^.

Therefore, it is essential to develop diagnostic tools that can detect mpox with high specificity and sensitivity, while allowing for rapid detection and easy distribution. There is a need for adequate tracking, reporting, and notification of households with suspected mpox cases in endemic regions to allow the population to practice isolation and to promote post-exposure prophylaxis. Effective mpox surveillance necessitates the widespread availability of PCR or sequencing testing, particularly in low-income households, to enable timely case tracking. Given the risk of reverse zoonosis, concurrent surveillance of animals in close contact with infected individuals is essential to prevent the establishment of reservoirs^[52]^. When mpox symptoms are suspected, the clinician should immediately report the cases, and in those cases where mpox is endemic, suspected and confirmed cases should be reported to WHO, Once the suspected case is confirmed to be mpox, identification, and tracing of all possible contacts should be done within the incubation period of mpox^[42]^.

## Future expectations

### Research directions

The current treatment regimen approved by the FDA includes tecovirimat, which has been previously used to treat smallpox. One trial showed that its use in mpox resulted in no side effects and that it led to a quicker improvement in lessons and a shorter illness span. A total of 7563 cases of patients receiving or prescribed antiviral drugs have been documented by the Centers for Disease Control. Despite these promising results, more rigorous clinical trials are necessary to confirm the efficacy and safety of this antiviral drug for mpox treatment.^[53-55]^

Preclinical studies, including animal models and in vitro experiments, have demonstrated the efficacy of cidofovir against various orthopoxviruses such as vaccinia, mousepox, and monkeypox^[55]^. Several studies have demonstrated the efficacy of cidofovir against MPXV. Although clinical data specifically for monkeypox treatment are limited, cidofovir remains a widely considered option based on its established antiviral properties. Thus, more evidence-based trials are warranted to assess the efficacy of cidofovir. Further studies are warranted in humans regarding the efficacy of brincidofovir against MPXVs^[41]^. Operational research on mpox is currently hindered by several challenges, including insufficient resources for comprehensive case investigations and contact tracing in the affected communities. The lack of adequate diagnostic facilities in laboratories poses a significant obstacle for accurate and timely diagnosis. These limitations contribute to difficulties in identifying the underlying etiologies and understanding the full extent of mpox transmission, including subclinical infections among contacts. A seroprevalence study would be instrumental in elucidating the epidemiology of mpox and in assessing the prevalence of asymptomatic cases within affected communities. To elucidate the transmission dynamics of other orthopoxviruses within human and animal populations, additional research employing molecular and genomic methodologies is required^[41]^

Mpox is a member of the genus Orthopoxvirus of the family *Poxviridiae* and possesses a linear double-stranded DNA genome encased by a lipid-containing dumbbell-shaped nucleocapsid. Genomic analysis of MPOX revealed that the predominant strain was the B.1 lineage of the West African clade. This strain exhibits several mutations in genes that are linked to virulence, host recognition, and immune evasion. Additionally, approximately 50 nucleotide polymorphisms have been isolated, and their functional significance remains unclear. This necessitates further research to elucidate the potential role of these mutations in MPOX transmissibility^[56]^.

The currently approved vaccines for Pox are ACAM2000 (Sanofi Pasteur Biologics Co.) and JYNNEOS(Bavarian NordicA/S). However, the mpox vaccination aspect is marked by global inequity owing to limited access by low- and middle-class countries. Vaccine efficacy data suggest that the smallpox vaccine is at least 85% effective against MPOX, and recent studies have shown the efficacy of JYNNEOS^[57]^. There is a need for targeted vaccination of high-risk groups, especially those countries with a higher mpox burden, to receive a larger share of the vaccine supply to reduce local transmission and prevent international transmission^[58]^.

A study found that there was a high level of willingness to use mpox vaccines and adherence to social isolation guidelines, indicating a strong understanding of public health measures. One of the main causes of disparity in vaccine distribution also comes from the limited supply, thus restricting it to high-risk groups, including people living with HIV, healthcare workers exposed to orthopoxviruses, and individuals with recent mpox exposure^[59]^.

### Global health implications

Due to the 2022 outbreak of Monkeypox following the recent outbreak of COVID–19, several countries have started taking immediate precautions such as investing in trials, buying vaccines, and increasing surveillance to control the epidemic. However, this has led to vaccine shortages and increased the burden on researchers to provide safe and efficacious treatment regimens with limited available data on mpox epidemiology. Given the World Health Organization’s declaration of monkeypox as a public health emergency of international concern, urgent action is required to enhance public awareness and diagnostic capabilities. Such measures are essential to mitigate the spread of the virus. Enhanced comprehension and clinical management of monkeypox, coupled with fortified infection prevention and control capabilities, particularly among public health professionals, are imperative. Concurrently, it is essential to effectively address stigma and discrimination within the monkeypox community, while ensuring equitable access to treatment and vaccines. A coordinated international effort is essential to expedite clinical trials evaluating the efficacy and safety of both monkeypox vaccines and antiviral drugs^[41]^.

MPOX has a significant impact on the physiology and psychology of the public and health sectors. One of its main consequences is painful skin lesions associated with the infection, which can result in scarring, thus leading to potential psychological trauma. Additionally, if the eye is infected by the virus, it can cause permanent visual impairment owing to corneal perforation^[56]^. This highlights the crucial need to promptly address skin lesions and prevent the spread of conjunctival secretions to contain the infection. There were evident global disparities in response to the mpox outbreak. A study in Nigeria found that 5.2% of healthcare workers were unaware of mpox, implying possible under-diagnosis when treating patients^[58]^. The WHO appointed an Emergency Committee in response to the mpox outbreak to declare mpox as a public health emergency of international concern (PHEIC) to prevent a delayed response to an outbreak, as seen in the case of the Covid-19 pandemic. There is a glaring inequality in which outbreaks in lower- and middle-income countries are often overlooked until they spread to high-income countries. Such delayed responses are concerning and must be addressed in future studies. There is a need for increased funding for disease surveillance, research, and the promotion of health equality. A global approach to each nation contributing to global preparedness efforts is essential^[60]^.

### Policy recommendations

ECDC provided the following recommendations^[53]^:

Mpox is an emerging epidemic that has been detected in blood, urine, tissue abscesses, and bodily fluids.

- Given the incubation period of mpox, asymptomatic donors who have had recent contact with confirmed cases should be deferred for at least 21 days after their last exposure.
- Confirmed or suspected mpox cases should be subjected to a 14-day deferral period for blood donation, commencing after the cessation of all symptoms.
- Recommended smallpox vaccination within 2 weeks, ideally 4 days, after significant, unprotected exposure to a diseased animal or a confirmed human case.

The spread of MPXV may be controlled by infected animals and by tracing their contacts.

Rigorous surveillance and public health interventions are essential to mitigate the spread of the mpox. This includes heightened monitoring of at-risk populations, such as travelers from affected regions and healthcare workers. Public education campaigns should emphasize the dangers of zoonotic transmission, particularly through bushmeat consumption, as well as the importance of personal hygiene and avoiding contact with infected individuals^[43]^.

Vaccination remains the most effective preventive measure, especially in active mpox transmission regions; therefore, it is imperative to enhance public awareness regarding vaccine efficacy while also mitigating their fears regarding its adverse effects to promote a stronger vaccination drive. Trifluridine eye drops are highly effective in infection affecting the eyes and this preventing the spread of infection^[56]^. There is a need to accelerate research efforts to develop effective strategies to combat outbreaks and the potential emergence of drug-resistant mpox strains that could be fatal. The most cost-effective method to prevent the spread of mpox is to implement personal prevention measures, such as avoiding contact with infected individuals, vaccination, and maintaining personal hygiene. There is a need to improve PCR analysis of specimens as a diagnostic tool, such as point-of-care diagnostic tools like Tetracore Orthopox Biothreat Alert, which can easily detect mpox with little expertise in endemic areas and require stronger laboratory facilities for testing and accurate reporting of cases^[61]^.

To effectively address Mpos without any disparity, it is crucial to implement active involvement of 2SLGBTQIAP+ in developing and implementing strategies that could improve access to testing and treatment. There is a need to eliminate societal stigma, foster a supportive environment, and challenge negative attitudes to combat the spread of mpox^[58]^.

## Conclusion

Mpox remains a significant global health concern, necessitating concerted efforts to curb its spread and to mitigate its impact. Strengthened surveillance, robust diagnostic capabilities, and targeted awareness campaigns are fundamental for addressing the current and future challenges posed by this disease. However, it is crucial to acknowledge the variability in the study quality across the available literature. Many findings are derived from observational studies or limited case series, which may introduce bias or limit the generalizability of the conclusions. A cautionary approach to interpret and apply these data is essential to avoid misinformed strategies. Awareness plays a pivotal role in the mitigation of mpox. Beyond informing the public about transmission routes and prevention measures, awareness campaigns can reduce stigma, particularly in disproportionately affected communities such as men who have sex with men and individuals with compromised immune systems. Reducing stigma is critical for encouraging timely reporting, seeking medical care, and improving adherence to public health measures. By integrating culturally sensitive education efforts with scientific advancements and equitable resource distribution, we can address the multifaceted challenges of mpox and improve preparedness for future outbreaks.

## Data Availability

Not applicable.
